# Differential regulation of *Pleurotus ostreatus* dye peroxidases gene expression in response to dyes and potential application of recombinant *Pleos*-DyP1 in decolorization

**DOI:** 10.1371/journal.pone.0209711

**Published:** 2019-01-04

**Authors:** J. Cuamatzi-Flores, E. Esquivel-Naranjo, S. Nava-Galicia, A. López-Munguía, A. Arroyo-Becerra, M. A. Villalobos-López, M. Bibbins-Martínez

**Affiliations:** 1 Centro de Investigación en Biotecnología Aplicada-Instituto Politécnico Nacional, Ex-Hacienda de San Juan Molino, Tepetitla de Lardizábal, Tlaxcala, México; 2 Facultad de Ciencias Naturales, Universidad Autónoma de Querétaro, Avenida de las Ciencias S/N Juriquilla, Querétaro, México; 3 Instituto de Biotecnología, Universidad Autónoma de México, Av. Universidad, Chamilpa, Cuernavaca, Morelos, México; Universidade Nova de Lisboa, PORTUGAL

## Abstract

Dye-decolorizing peroxidase (DyP) from the white rot basidiomycete *Pleurotus ostreatus* is a heme peroxidase able to oxidize diverse substrates, including recalcitrant phenols and dyes. This study analyzed the effect of chemical dyes on *P*. *ostreatus* growth, DyP activity and the expression of four *Pleos-dyp* genes during the time-course of *Pleurotus ostreatus* cultures containing either Acetyl Yellow G (AYG), Remazol Brilliant Blue R (RBBR) or Acid Blue 129 (AB129) dyes. Additionally, Pleos DyP1 was heterologously expressed in the filamentous fungus *Trichoderma atroviride* in order to explore the potential of a secreted recombinant enzyme for decolorizing different dyes in cultures and plate assays. The addition of dyes had an induction effect on the enzymatic activity, with the fermentations undertaken using RBBR and AYG dyes presenting the highest total DyP activity. DyP gene expression profiles displayed up/down regulation during the culture of three *Pleos-dyp* genes (*Pleos-dyp1*, *Pleos-dyp2* and *Pleos-dyp4*), while *Pleos-dyp3* transcript was not detected under any of the culture conditions studied. A 14-fold relative induction level (log2) increase for *Pleos-dyp2* and *Pleos-dyp4* in AB129 and AYG, respectively, was also found. The presence of AB129 resulted in the highest *Pleos-dyp1* gene induction and repression level, corresponding to 11.83 and -14.6-fold relative expression and repression levels, respectively. The lowest expression level of all genes was observed in RBBR, a response which is associated with the growth phase. The filamentous fungus *Trichoderma atroviride* was successfully transformed for the heterologous expression of *Pleos-dyp1*. The modified strains (TaDyP) were able to decolorize mono-azo, di-azo, anthraquinone and anthracenedione dyes with extracellular DyP1 activity found in the culture supernatant. After 96 h of culture, the recombinant TaDyP strains were able to degrade (decolorize) 77 and 34% of 0.05mM AB129 and 0.25mM AYG, respectively.

## Introduction

Dye-decolorizing peroxidases (DyPs; EC 1.11.1.19) were first purified and characterized in 1999, in the basidiomycete *Bjerkandera adusta* Dec 1 [1,2]. They are heme peroxidases, and their name reflects their ability to degrade several anthraquinone dyes, with the heme group used as redox cofactor to catalyze the hydrogen peroxide-mediated oxidation of many kinds of molecules, including dyes. To date, these enzymes have been identified in the genomes of fungi, bacteria, and archaea, although their physiological function is still unclear [3].

Phylogenetic tests have revealed four DyP subfamilies (A-D), with fungal DyPs found mainly in Subfamily D, indicating that they may have evolved via horizontal gene transfer (HGT) from cyanobacterial predecessors [4].

The first gene, and corresponding cDNA, that encodes a DyP in *Pleurotus ostreatus* was first identified on the basis of sequence homology analyses [5], which revealed that the amino acid sequence of the gene corresponding to Pleos-DyP1 shares 43% of its identity with DyP from the basidiomycete *Bjerkandera adusta* Dec 1. Four years later, a search for the nucleotide sequences of heme peroxidases in the *Pleurotus ostreatus* genome and a structural-functional analysis of the heme peroxidases found four different models of the DyP peroxidase-type family [6]. Two out of the four DyP genes identified were then analyzed and classified as phylogenetically divergent (Pleos-*dyp1* and Pleos-*dyp4*). The heterologous expression of Pleos-*dyp1* and Pleos-*dyp4* and the characterization of the corresponding enzymes Pleos-DyP1 and Pleos-DyP4 were later undertaken, in which different catalytic and stability properties for both enzymes were observed. Moreover, it was reported that Pleos-DyP4 shares catalytic properties with a versatile peroxidase enzyme (VP), while secretomic studies performed revealed that only the Pleos-DyP4 enzyme, as well as several VPs and MnPs, forms part of the *P*. *ostreatus* extracellular proteome during growth on lignocellulosic materials [7].

*Escherichia coli* is one of the most widely used hosts for the production of eukaryotic recombinant proteins. However, recurrent problems in the recovery of substantial yields of correctly folded and active enzymes have been found in the particular case of *P*. *ostreatus* proteins [8]. While several fungal DyPs have been expressed in *E*. *coli*, different strategies for protein recovery, from inclusion bodies, refolding, and in-vitro purification, have been required, thus decreasing productivity and increasing production costs. On the other hand, *Trichoderma* species have been reported as efficient systems for the heterologous expression of proteins due to their potential for carrying out eukaryotic post-translational modifications and efficient secretion [9].

This study reports the differential expression of *P*. *ostreatus dyp* genes (*dyp1*, *dyp2*, *dyp3* and *dyp4*) in a media supplemented with different dyes, the heterologous expression of the gene encoding DyP1 in *Trichoderma atroviride*, and its effect when applied to dyes.

## Materials and methods

### Microorganisms

This research used *Pleurotus ostreatus* from the American Type Culture Collection (ATCC 32783) (Manassas, Virginia, U.S.A.) The strain was grown and maintained in potato dextrose agar (PDA).

*Trichoderma atroviride* IMI206040 was used as the wild-type strain for carrying out the heterologous expression of the protein of interest. Wild-type or transformed strains were propagated in PDA medium at 28°C, with hygromycin B (100 μg/mL) added when necessary.

*Escherichia coli* XL-blue was used as a host strain for cloning vectors and the propagation and purification of plasmid. The *E*. *coli* was grown in LB medium at 37°C to which, when required, ampicillin (100 μg/mL) or chloramphenicol (34 μg/mL) were added.

### *P*. *ostreatus* liquid cultures

Both the composition of the medium and the conditions for the submerged cultures were adapted from Garrido-Bazan, et al., [10]. Four fermentations of *P*. *ostreatus* grown in control medium (CMF) in the presence of either 500 ppm of remazol brilliant blue R dye (RBBR) (dye content: 50%) (SIGMA-ALDRICH R8001), 500 ppm of Acetyl yellow G (AYG) (dye content 95%) (SIGMA-ALDRICH 250309), or acid blue 129 (AB129) (dye content 25%) (SIGMA-ALDRICH 306495) were generated for this study. Each flask was inoculated with three mycelial plugs (4 mm diameter) taken using a steel punch from the periphery of *P*. *ostreatus* colonies grown for 7 d at 25°C in Petri dishes containing potato dextrose agar. The cultures were incubated at 25°C for 23 days on a rotary shaker (SEV-PRENDO 650M) at 120 rpm. Three flasks were taken as samples at 120, 144, 168, 216, 240, 264, 288, 312, 336, 360, 408, 480, 504 and 552 h of fermentation. The enzymatic extract (EE) was obtained by filtering the cultures using Whatman No. 4 filter paper and then stored at -20°C until it was analyzed, while the mycelium was rinsed with 0.9% NaCl and stored at -70°C until subjected to the total RNA extraction procedure or used for biomass (X) determination via dry weight measurement (g/L). The decolorization of the dyes was monitored spectrophotometrically at each dye’s maximum absorbance. All experiments were performed in triplicate.

### RNA extraction and RT-qPCR

The total RNA was isolated from frozen mycelia harvested at different fermentation times, using the NTES extraction protocol, and was spectrophotometrically quantified by determining the absorbance ratio at OD 260/280. The RNA was treated with RNAse-free DNase I (Invitrogen). The final RNA concentration was set to 300 ng/μl, after which 3 μg of total RNA was reverse-transcribed into cDNA in a volume of 20μl using M-MuLV Reverse Transcriptase (Fermentas), following the manufacturer’s protocol.

The RT-qPCR reactions were performed in a StepOne Plus thermal cycler (Applied Biosystems), using SYBR green dye to detect the amplification of product. Specific primers were design for amplifying the transcript from the four *Pleos-dyp* genes identified in the genome (Table 1). The reaction mixture, the amplification program and the melting curve were adapted from Garrido-Bazan, et al., [10], while all RT-qPCR reactions were carried out in triplicate with a template-free negative control performed in parallel.

**Table 1:** Primers used in this study.

Gene	Purpose of use	Transcript ID^a^	Direction^b^	Sequence (5’ to 3’)	Product size (bp)	Efficiency^c^
*gpd*	qPCR	1090672	R	GTGCAAGACGCATTTGAG	83	2.00
			F	GCTGACGCACCAATGTTC		
*Pleos_dyp1*	qPCR	62271	F	CGCTTGAGTTGATCCAGAAA	104	2.21
			R	TATTTCCTTCGGCTTCCTCA		
*Pleos*_*dyp2*	qPCR	1092668	F	TACATTCTTGCCGCTGGAT	117	1.87
			R	GCGAGAACCTGCTTGAACTT		
*Pleos*-*dyp3*	qPCR	52170	F	CCGAAGGTTCAAGCAAGT	80	nd
			R	GGCAAGTACCGCAGATAAG		
*Pleos*_*dyp4*	qPCR	1069077	F	ATGAACACTTCGGCTTCCTC	64	2.03
			R	GGGTTCTGGTCGAATTGC		
*Pleos*_*dyp1*	Cloning^d^	62271	F	^**e**^***GCGGCCGC***TACCAACTCATCTACAATGC	1581	
			R	^**f**^***AAGCTT***AAGCAGCGATTTTGTGCAAGATG		
*Hph* R^g^	Cloning	-	F	GATCGACGTTAACTGATATTGAAGGAG	1420	-
			R	CTATTCCTTTGCCCTCGGACGAGTGCTG		-

Note

^a^Transcript ID and gene nomenclature refer to the annotation of *P*. *ostreatus* PC15—genome version 2.0 (http://genome.jgi-psf.org/PleosPC15_2/PleosPC15_2.home.html)

^b^ Fw—Forward, Rv—Reverse

^c^ Efficiency for primers used solely in qPCR

^d^ To amplify the ORF gene in cDNA

^e^ NotI site

^f^ HindIII site

^g^ Detection of integrative vector; nd—Non detected

The PCR efficiency (*E*) was calculated using the slope of the standard curve according to the equation E = 10^[−(1/slope)]^ [11].

### Reference genes and statistical analyses

A previous study by Garrido-Bazan, et al., [10] supported the reference genes used under these conditions. The data processing of the results was initially performed using Microsoft Excel 365 and included efficiencies and reference gene normalization, while the fold expression was calculated by the 2^-Δ ΔCt^ method, as described by Pfaffl [11] (Eq 1).

$$ratio=\frac{{\left(E_{target}\right)}^{{\Delta CP}_{target}(control-sample)}}{{\left(E_{ref}\right)}^{{\Delta CP}_{ref}(control-sample)}}$$ Eq 1

In the above equation, E_target_ is the real-time PCR efficiency of the target gene transcript, while E_ref_ is the real-time PCR efficiency of a reference gene transcript, and ΔCp_target_ is the CP deviation of the control sample of the target gene transcript.

### Cloning and heterologous expression

The native *Pleos*_DyP1 cDNA from *P*. *ostreatus* was amplified by PCR using the High Fidelity PCR Enzyme Mix (Fermentas) and a set of cloning primers that were designed based on the sequence JGIgenome 62271(https://genome.jgi.doe.gov) (Table 1). The product of the above amplification was cloned into the vector pJET1.2 blunt (Thermo Fisher Scientific) for further sub-cloning in the integrative expression vector pUE10 [12]. This vector has resistance genes used for the selection of hygromycin and chloramphenicol in *T*. *atroviride* and *E*. *coli*, respectively. The blu17 terminator and the sequence downstream enable this construction to be integrated into a locus near the blu17 terminator intergenic region of *T*. *atroviride*. *Pleos*_DyP1 open reading frame (ORF) was cloned downstream of the pyruvate kinase promoter (P*pki*) using NotI and HindIII restriction sites. Several clones were analyzed via enzymatic digestion, with the positives then verified through sequencing.

The transformations undertaken in this research were carried out as described previously [13], with the transformed protoplast spread and incubated, in the presence of light, on PDA plates containing hygromycin B, until spores were obtained. These spores were re-plated for at least three times until stable lines were obtained. The full-length insertion of the vector into the genome of the transformants was confirmed via genomic PCR.

### Dye decolorization assays

Fresh spores collected from cultures growing on PDA for one week at 28°C in the presence of light and counted in a Neubauer chamber were used for evaluating the growth capacity of over-expressing *Trichoderma atroviride* strains in PDA with synthetic dyes. Drops of each spore suspension, containing either 500 conidia of WT or over-expressing strains, were placed on PDA supplemented with 0.5% Triton X-100 and the following dyes: acetyl yellow G; remazol brilliant blue R; acid blue 129; and, reactive black 5. All dyes were used at a final concentration of 100 ppm and all experiments were carried out in triplicate.

Two strains with similar on-plate growth and decolorization rates were chosen to evaluate the decolorization capacity on submerged fermentations, while 500 spores of WT and over-expressing strains were placed on PDA without Triton-X100 and with the above described dyes for 96 h.

For the submerged fermentations, PDB medium was inoculated with 1x10^6^ spores in 50 mL of medium supplemented with 100 ppm of either AYG or AB129 dyes, under growth conditions of 28°C and 180 rpm for 96 h. The enzymatic extract (EE) was obtained via the filtration of the cultures using Whatman No. 4 filter paper, while the fungal mycelium was used for determining the biomass (X) via the measurement of dry weight (g/L). The kinetic growth was established via dry weight measurement and total protein was measured in accordance with Bradford. The decolorization of the dyes used was monitored with a UV/Vis spectrophotometer. The experiments were performed in triplicate, with the values shown representative of at least two of the experiments.

### Determination of DyP activity

The dye decolorizing peroxidase activity was determined as described previously [14], with the reaction incubated at 45°C for 1 min, and expressed in international units (U/mL). One international unit (1 UI) was defined as the amount of enzyme oxidizing 1 μmol of substrate per minute.

## Results

This research analyzed the expression of the four *Pleos-dyp* genes via real time RT-qPCR during the time-course of *Pleurotus ostreatus* cultures containing the AYG, RBBR or AB129 dyes. Additionally Pleos DyP1 was heterologously expressed in the filamentous fungus *Trichoderma atroviride* and the potential of the modified strain for decolorizing different dyes on plate assays and cultures then evaluated.

### *Pleurotus ostreatus* growth and DyP activity in liquid culture

**Fig 1:**
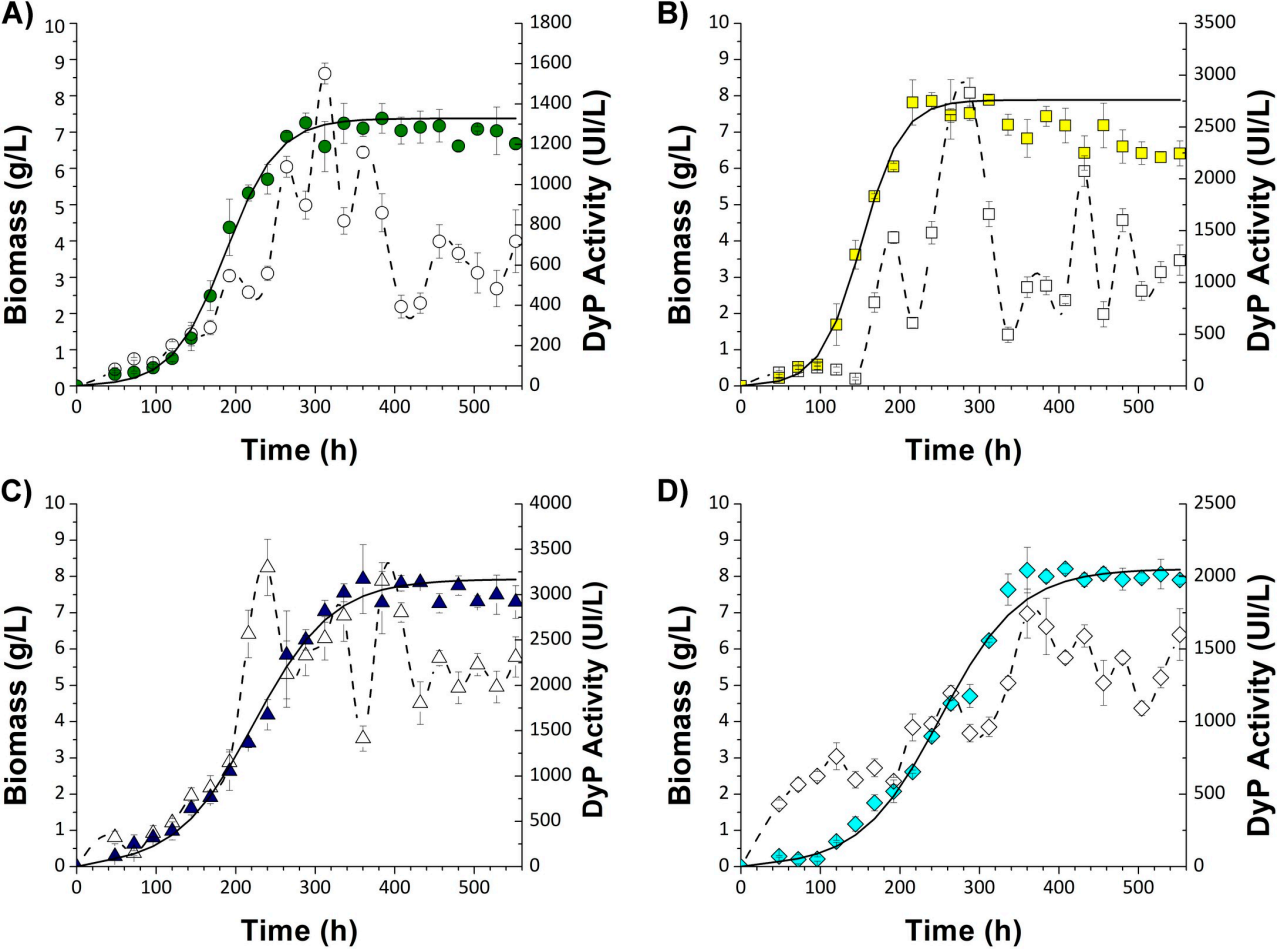
Growth of *Pleurotus ostreatus* and Pleos-dyP activity. *Pleorutus ostreatus* was grown on submerged fermentation in a control media **(A)** and in the presence of 500 ppm of different dyes–**(B)** AYG, **(C)** RBBR, and **(D)** AB129. Time course of extracellular DyP activity (open symbols) and *P*. *ostreatus* biomass (colored closed symbols). Bars show standard deviation.

The growth of *P*. *ostreatus* and total Pleos-DyP activity in both the control culture and cultures containing the dyes are shown in Fig 1. The addition of dyes did not affect the maximum production of biomass (X_max_), which did not show significant differences with the X_max_ obtained for the basal fermentation. However, the specific growth rate (μ) varied with each type of dye, ranging from 0.038 h^-1^ for AYG, 0.02 h^-1^ for AB129 and 0.019 h^-1^ for RBBR fermentations, compared to the 0.03 h^-1^ observed in the control fermentation. All fermentations supplemented with dyes presented maximum activity values that were higher than those obtained for the control fermentation. The maximum activity during the control fermentation was 1550 IU/L at 312 h, compared to 3298, 2828 and 1744 IU/L for the RBBR, AYG and AB129 fermentations, at 240 h, 288 h and 360 h, respectively. On the other hand, a rapid transformation of 500 ppm was observed for all dyes between 48 and 144 h (100% decolorization for RBBR (0.39 mM) and 70% decolorization for both AYG (1.25 mM) and AB129 (0.27 mM)). Although the decolorization of AYG and AB129 occurred at a slower rate, the total removal of both dyes (100% decolorization) was achieved after 552 h (S1 Fig).

### Differential expression profiles of *Pleos*-*dyp* genes in response to dyes

**Fig 2:**
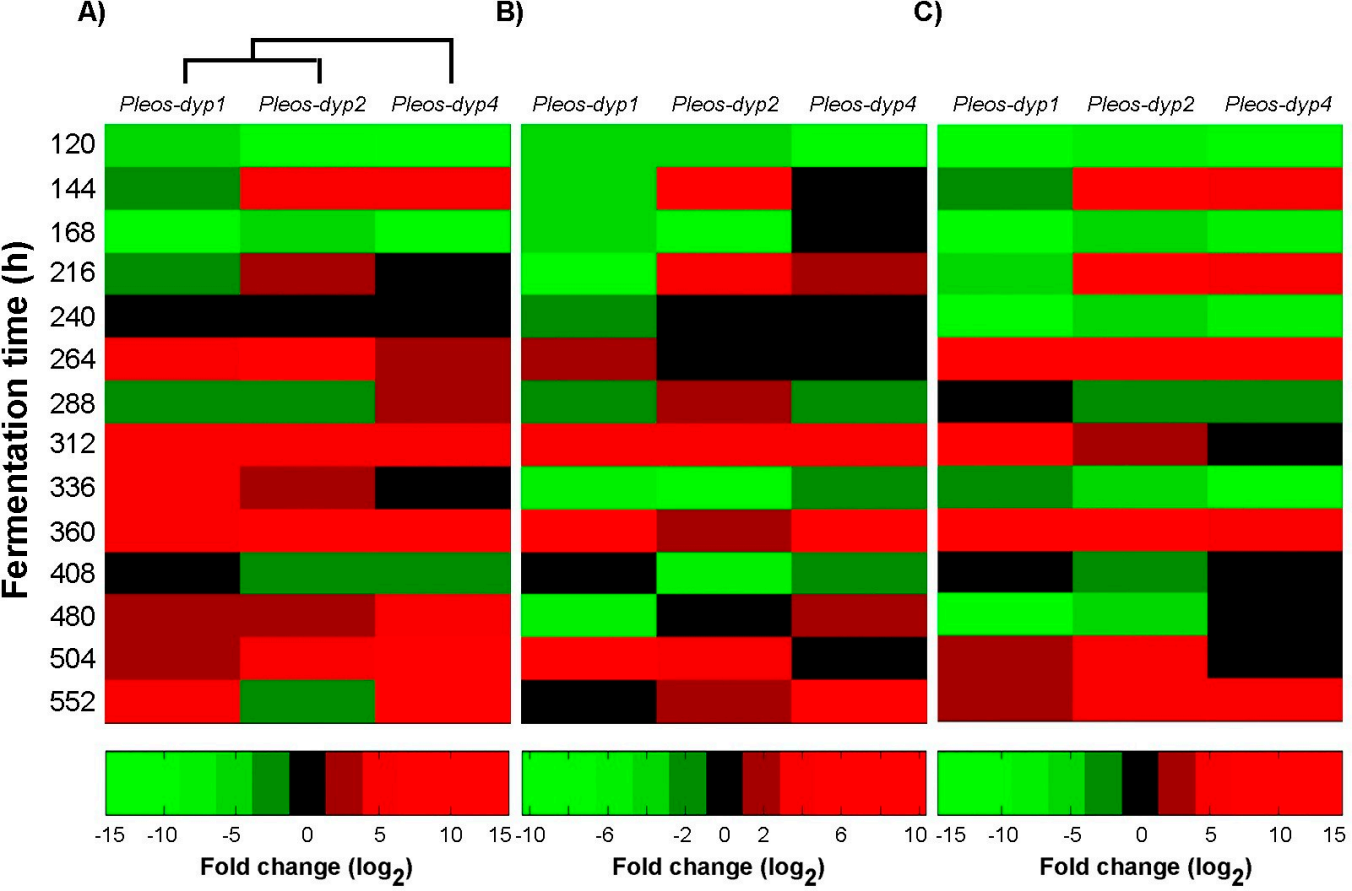
Heat map of global gene expression. Exploratory analysis of the gene expression of three *Pleos-dyp* genes during the time-course of the submerged fermentation of *Pleurotus ostreatus* containing **(A)** AYG, **(B)** RBBR and **(C)** AB129 dyes.

The expression of *Pleos-dyp1*, *Pleos-dyp2* and *Pleos-dyp4* genes in response to the addition of synthetic dyes was evaluated using RT-qPCR. The expression profiles displayed as a heat map associated with a dendrogram (Fig 2) classified the *Pleos-dyp1* and *Pleos-dyp2* expression profiles as similar, in contrast to that obtained for the *Pleos-dyp4* gene.

**Fig 3:**
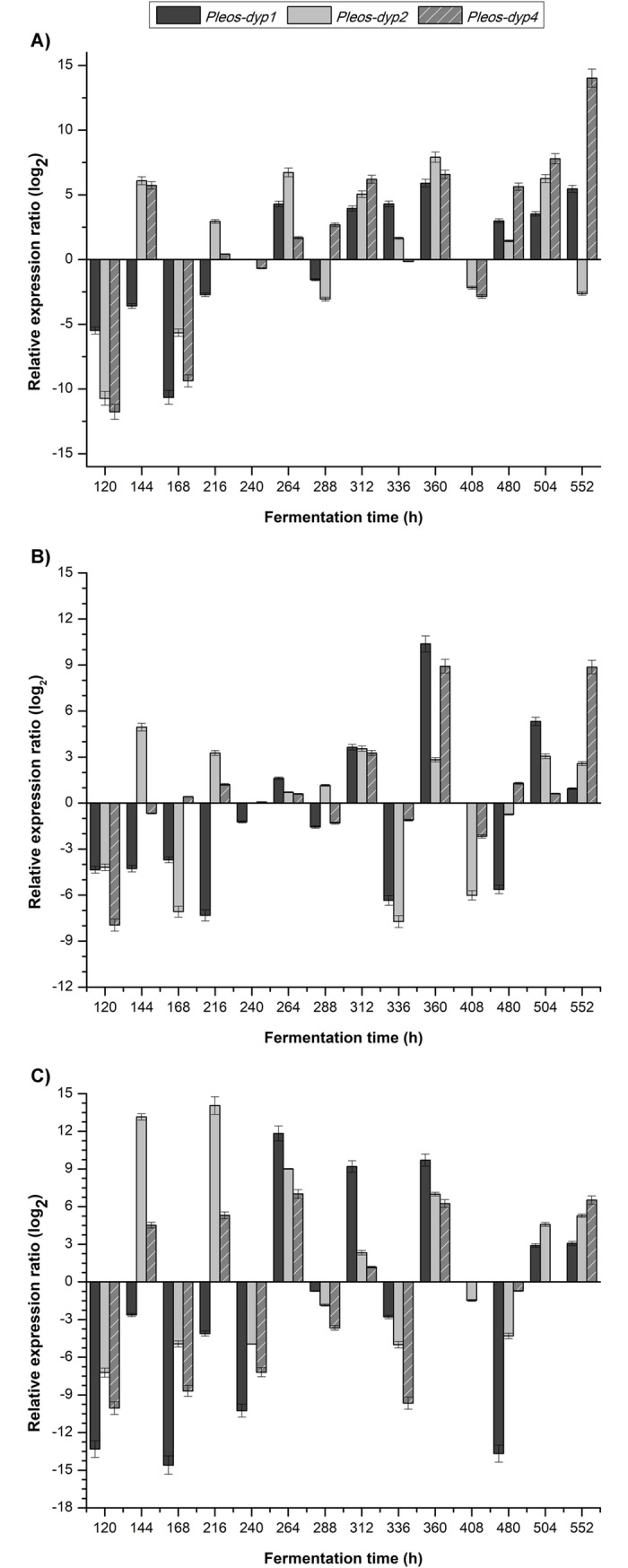
Effect of dyes on the relative expression levels of the three DyP genes. The expression patterns of the genes *Pleos-dyp1*, *Pleos-dyp2* and *Pleos-dyp4* in response to the addition of synthetic dyes during the time-course of a submerged *Pleurotus ostreatus* fermentation with **(A)** AYG, **(B)** RBBR and **(C)** AB129. The increased or decreased expression values were referred to those obtained under the control condition (without dye). Error bars represent the standard deviations of the means of three independent amplifications.

In all conditions, the differential expression patterns of the three *dyp* genes in the different dyes were growth phase dependent. In general terms, the expression profiles of the three *dyp* genes (*Pleos-dyp1*, *Pleos-dyp2* and *Pleos-dyp4*) displayed up/down regulation along the culture, while the *Pleos-dyp3* transcript was not detected under any of the culture conditions (Fig 3). A 14-fold relative induction level (log2) increase was found at 216 h for *Pleos-dyp2* and at 552 h for *Pleos-dyp4* in AB129 and AYG, respectively. The presence of AB129 resulted in both the highest *Pleos-dyp1* gene induction and repression levels found in this research, corresponding to 11.83 and -14.6-fold relative expression and repression levels at 264 h and 120 h, respectively. The lowest expression level of all the genes was observed in RBBR, for which, *Pleos-dyp1* presented the highest induction level, with a relative induction level of 10.37 at 360 h. All three genes showed a highest repression level corresponding to a -7.95 fold change at 120 h.

### Heterologous expression of Pleos-dyP1 in *T*. *atroviride*

Based on the decolorization levels and expression profiles found throughout the different dye cultures, the *Pleos-dyp1* gene was selected for its subsequent heterologous expression and then successfully cloned and expressed in the filamentous fungus *Trichoderma atroviride*. In the first step, an expression vector (pUE10) designed by Balcazar-López, et al., [12] was used to clone *Pleos-dyp1*, the result of which was then used to transform *T*. *atroviride*. The vector pUE10 enables, via homologous recombination, the integration of the gene of interest into an intergenic locus near the blu17 terminator region of *T*. *atroviride*. After the execution of three monosporic cultures, fifteen transformants with resistance to hygromicin B were obtained, all of which were verified by PCR to ensure that they had been constructed correctly. The capacity of the fifteen transformants to grow and decolorize mono-azo (AYG), di-azo (RB5), anthraquinone (RBBR) and anthracenedione dyes (AB129) was initially evaluated on PDA plates containing 0.2 mM of each dye. All the transformant strains (TaDyP1) obtained were able to decolorize the dyes tested in this study. Two of them (TaDyP1_7 and TaDyP1_12) were selected for further study due to their similar growth rate and DyP activity.

**Fig 4:**
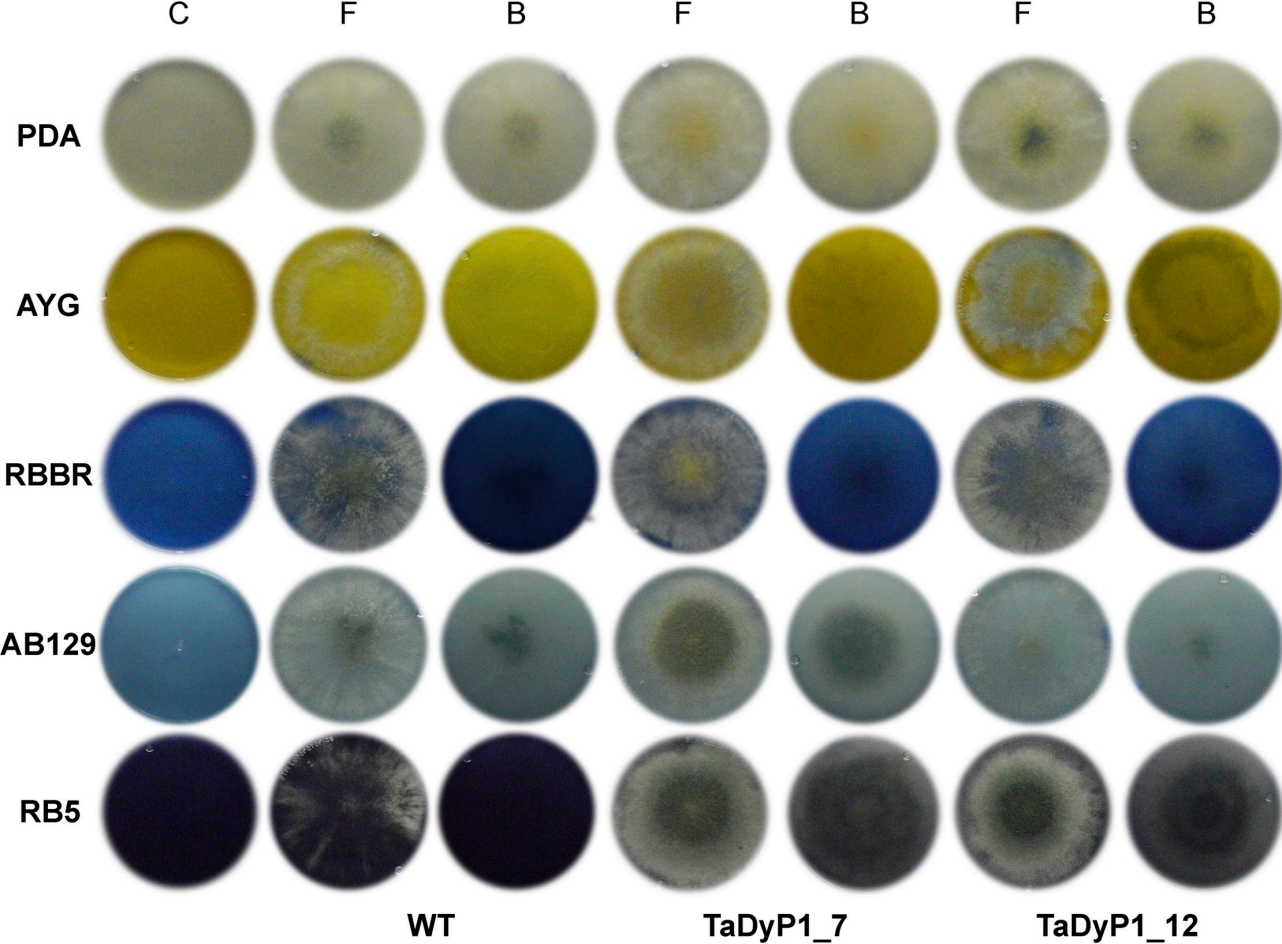
Decolorization of synthetic dyes by *T*. *atroviride* transformants. The over-expressing Pleos-DyP1 strains selected were grown on PDA in the presence of 100 ppm of AYG, RBBR, AB129 or RB5 dyes at 28°C for 96 h. Wild-type **(WT)**, *T*. *atroviride* transformants **(TaDyP1_7 and TaDyp1_12)**, control without inoculum **(C)**, petri dish front **(F)**, petri dish back **(B)**.

The two strains selected were then grown on PDA containing 100 ppm of the four dyes previously described (Fig 4), finding that, while the wild-type *T*. *atroviride* strain grows in this media, it is not capable of decolorizing the dyes. In contrast, both transformant strains selected were able to grow in this media and decolorize all dyes to a large extent, with the best decolorization results observed for the RB5 and AB129 dyes.

### Production of heterologous *Pleos*-dyP1 and evaluation of dye decolorization kinetics in liquid culture

**Fig 5:**
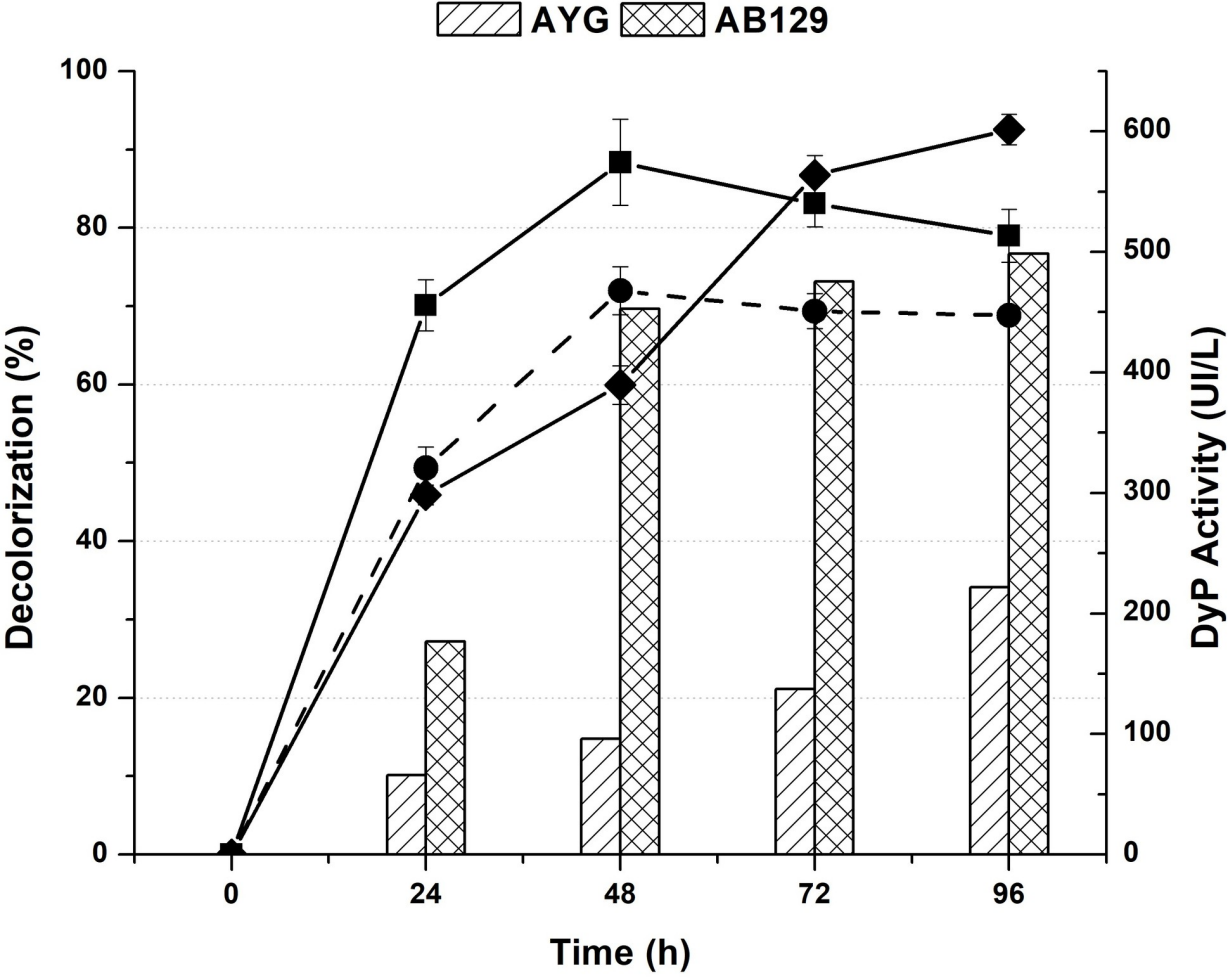
Time course of recombinant DyP activity and percentage dye decolorization. The evolution of the extracellular DyP activity and percentage dye decolorization during the liquid cultures of the *T*. *atroviride* TaDyP1_7 transformant was determined in both PDB medium and PDB containing either the AYG or AB129 dyes. DyP activity (broken line) in PDB (●black circle), AYG (■ black square) and AB129 (♦ black diamond). Data points represent the mean of three independent replicates, while the standard error of the mean (SE) is indicated by error bars.

**Fig 6:**
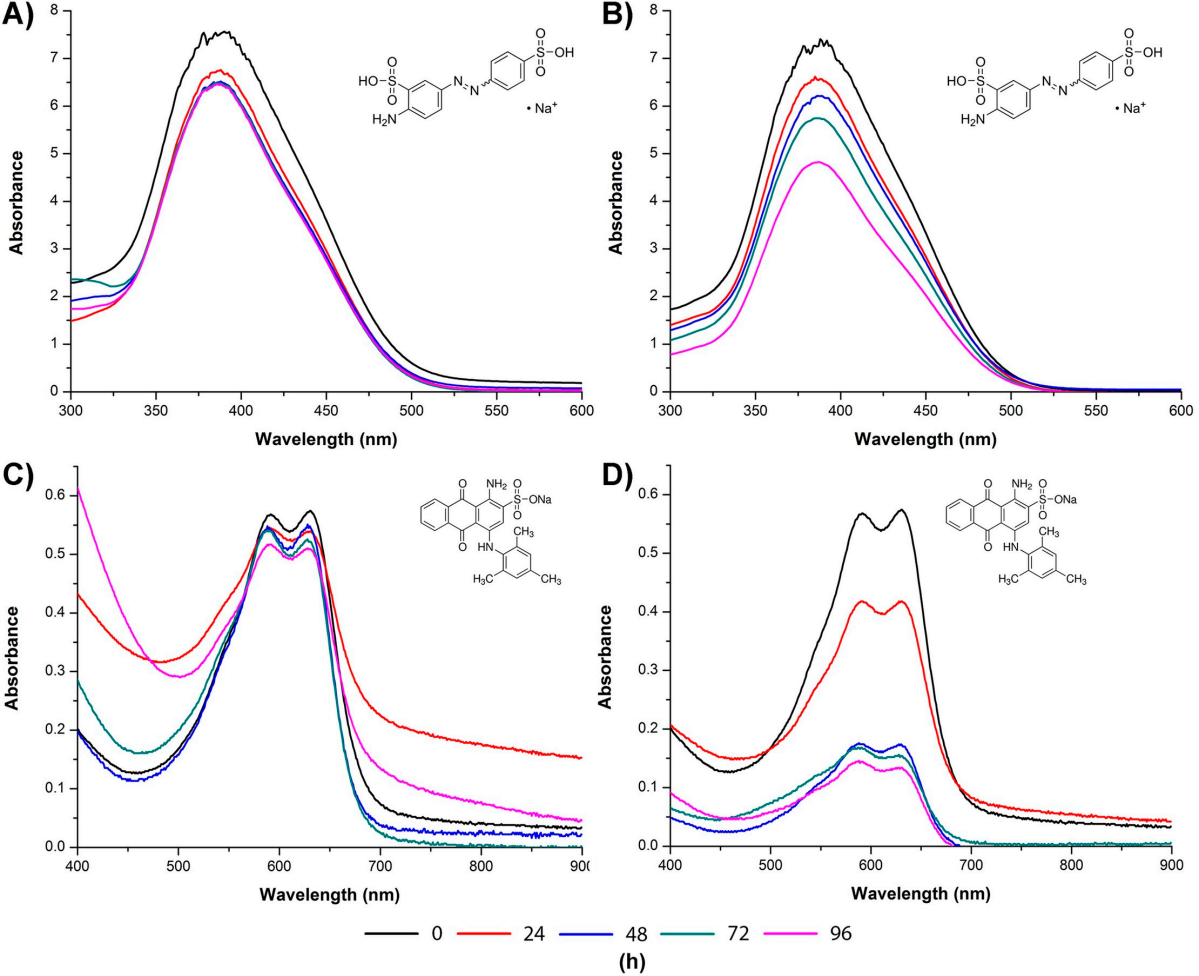
UV/Vis spectral changes of dyes. The UV/Vis spectra variation for the AYG and AB129 dyes was monitored during the *T*. *atroviride* culture-course, conducted at 28°C for 96 h. **(A)** WT strain in AYG, **(B)** TaDyP1_7 strain in AYG, **(C)** WT strain in AB129 and **(D)** TaDyP1_7strain in AB129. Maximum absorbance for AYG at 390 nm and AB129 recorded at 630 nm. The inset structural formula in A-B is AYG and AB129 in C-D.

The TaDyP1_7 strain was selected for further evaluation after growth in PDB liquid medium (basal) and PDB liquid medium supplemented with either AYG or AB129. The growth parameters, maximal biomass produced (Xmax), and specific growth rate (μ) were determined and are shown in Table 2, while Figs 5 and 6 show the dye peroxidase activity and the percentage of decolorization measured during the culture.

**Table 2:** Growth parameters of the WT and TaDyP1_7 strains in liquid culture.

Strain Culture	WT		DyP1_7	
	μ (h^-1^)	X_max_ (g/L)	μ (h^-1^)	X_max_ (g/L)
**PDB**	0.097	7.52	0.095	7.92
**AYG**	0.094	7.88	0.094	8.96
**AB129**	0.098	7.39	0.105	8.76

Compared to the basal condition, for both the WT and TaDyP1_7 strains, the specific growth rate (μ) and maximal biomass were not significantly affected in the cultures containing dyes. It should be noted that the X_max_ for the transformant strain was higher than the WT X_max_ in all conditions tested.

The DyP1 activity in the *T*. *atroviride* transformant was also found in the extracellular culture extract. Fig 5 shows the DyP enzymatic activity and decolorization percentage during the culture-course with the *T*. *atroviride* TaDyP1_7 transformant. For the WT strain, no dye peroxidase activity was detected in any of the conditions assayed (results not shown). Furthermore, for the TaDyP1_7 cultures, the highest activities were 601.5 UI/L at 96 h, 574.9 UI/L at 48 h and 467.9 UI/L at 48 h, for AB129, AYG and PDB, respectively. The decolorization percentage for both dyes increased gradually over the culture-course, reaching percentages of 77% and 34% discoloration after 96 h for AB129 and AYG, respectively.

The UV/Vis changes in absorption spectra for the dyes (AYG and AB129) during the *T*. *atroviride* culture-course are shown in Fig 6. The TaDyP strain (Fig 6B and 6D) showed spectral changes which coincide with the decolorization percentages. Such changes are evidence of the decolorization process undertaken by the DyP enzyme. The absorption spectra for the cultures with the WT strain did not present significant changes during the culture-course.

## Discussion

### Differential expression of *Pleos*-*dyp* genes in response to dyes

#### DyP in *P*. *ostreatus*

The existence of gene families that encode ligninolytic enzymes is very common in white-rot basidiomycetes [6,15–17]. According to Ruíz-Dueñas, et al., [6], there are four genes annotated on the *P*. *ostreatus* genome that encode DyP enzymes (*Pleos-*DyP-1 to *Pleos-*DyP-4). Sequence analysis of these genes reveals differences in the number of introns and also in the presence of signal sequences that guide their transit through both the endoplasmic reticulum and Golgi apparatus, suggesting different cellular locations or secretion pathways. Moreover, a phylogenetic analysis of all DyP sequences from the *Agaricomycotina* genomes available classified *Pleos*-DyP1, *Pleos*-DyP2 and *Pleos*-DyP3 into Cluster I, which includes all the DyPs identified from the orders *Auriculariales*, *Gomphales*, and *Sebacinales*. However, only *Pleos*-DyP4 was classified in Cluster III, which is mainly constituted by sequences from *Agarycales* and *Polyporales*. In addition, only two out of four *Pleos*-DyP genes were found to be phylogenetically divergent (*Pleos-*DyP1 and *Pleos-*DyP4) [7].

This research analyzed the effect of dyes on *P*. *ostreatus* growth, DyP activity and the expression of the four encoding genes for *Pleurotus ostreatus* dye-decolorizing peroxidase (*Pleos-dyp* genes) during the time-course of a submerged *P*. *ostreatus* fermentation supplemented with Acetyl Yellow G (AYG), Remazol Brilliant Blue R (RBBR) or Acid Blue 129 (AB129) dyes. Under these conditions, the addition of dyes did not affect the maximum production of biomass (X_max_); however, the specific growth rate (μ) varied with each type of dye. Moreover, the addition of dyes had a clear induction effect on the enzymatic activity of DyP. Fermentations with the RBBR and AYG dyes showed the highest total DyP activity. Under the same fermentation conditions used in the present research, Garrido-Bazan, et al., [10], reported that the RBBR and AYG dyes had also an induction effect on laccase activity, which almost doubled for both dyes in comparison with the control fermentation. However, maximum laccase activity was produced at the end of the stationary growth phase (480 h-576 h), a finding which contrasts with the maximum DyP activity, which occurred at the end of the exponential phase and the beginning of the stationary phase (240h-360 h).

The correlation among expression profiles (Fig 3), DyP activity (Fig 1) and decolorization levels during the time course of the different *P*. *ostreatus* fermentations (S1 Fig) shows that the highest levels of induction for *Pleos-dyp1* occur at the stage where the highest levels of enzyme activity and decolorization are presented (144–360 h), in contrast with the highest levels of induction for *Pleos-dyp4*, which occur during the late stages of fermentation (360–552 h). This may indicate that Pleos-DyP1 or Pleos-DyP2 could be the main DyP enzymes participating in the early decolorization of the dyes studied in the present research.

The *Pleos*-*dyp* gene expression profiles for three DyP genes displayed up/down regulation along the fermentation time (*Pleos-dyp1*, *Pleos-dyp2* and *Pleos-dyp4*), while *Pleos-dyp3* transcript was not detected in any of the fermentation conditions. In addition, the analysis of the expression profiles, displayed as a heat map associated with a dendrogram (Fig 2), classified the *Pleos-dyp1* and *Pleos-dyp2* expression profiles as similar, which differs from those expression profiles obtained for the *Pleos-dyp4* gene, suggesting the co-regulation of *Pleos-dyp1* and *Pleos-dyp2* in the presence of the dyes.

The highest relative induction level (log2) was a 14-fold change for *Pleos-dyp2* and *Pleos-dyp4* in AB129 and AYG, respectively. The addition of AB129 caused the highest induction and repression levels for the *Pleos-dyp1* gene, with relative expression and repression levels of 11.83 and -14.6 fold changes, respectively. The expression level for all genes in the presence of RBBR was lower than that found for AYG and AB129, a response which was growth phase dependent in all conditions.

To date, no report on the transcription regulation of *dyp* genes has been published; however, it is known that the differential expression of ligninolytic peroxidase genes in *P*. *ostreatus* and other fungi depends on conditions such as the environmental, nutritional and developmental stage [17–20]. Therefore, all the dyes tested in this research modified the expression profiles of *Pleos-dyp* genes, with a 14-fold change maximum induction level detected for *dyp* genes in the AYG and AB129 fermentations. Under the same fermentation conditions, maximum laccase expression levels comprising 6.8 and 4.6 fold changes at 408 and 144 h for AYG and RBBR, respectively, have been reported [10]. This contrasts with the maximum dye-decolorizing peroxidase expression levels of 14 and 10.3 fold-changes at 216 h and 360 h for AYG and RBBR, respectively, detected in this research. This suggests that, in the process of dye mineralization by *P*. *ostreatus*, the regulation of the ligninolytic system occurs in cascade, with some oxidases participating first, followed by the others.

It has been reported that the ligninolytic isoenzymes coded by members of a gene family often present differences in terms of their differential expression, catalytic properties, regulation mechanisms and location [10,17,20–22]. Promoter analysis of the ligninolytic enzymes encoding genes in the *P*. *ostreatus* genome shows the presence of different putative responsive elements [17,22,23], such as heat-shock elements (HSE), metal-response elements (MRE), xenobiotic-response elements (XRE) and cAMP response elements (CRE), which may be involved in the regulation of their expression by environmental conditions.

### Heterologous expression

Two of the four Pleos-DyP genes (*Pleos*-DyP1 and *Pleos*-DyP4) have been analyzed by heterologous expression [7,24]. Fernández-Fueyo, et al., [7] carried out the heterologous expression and purification of *Pleos*-DyP1 and *Pleos*-DyP4 synthetic sequences in *E*. *coli* BL21 with a plasmid pET23a. They reported that the heterologously expressed enzymes were able to oxidize anthraquinoid dyes (such as Reactive Blue 19), low redox-potential dyes (such as 2,2-azinobis-(3-ethylbenzothiazoline-6-sulfonic acid)) and substituted phenols. However, only Pleos-DyP4 oxidizes the high-redox-potential dye Reactive Black 5, at the same time displaying high thermal and pH stability.

A study by Behrens, et al., [24] also reported the heterologous expression of *P*. *ostreatus Pleos*-DyP1 and three DyP genes–taken from *A*. *auricular-judae* (AajDyP), *B*. *adusta* (BaDyP) and *M*. *scorodonius* (MsP2)–in *Escherichia coli*, using the cold shock-inducible expression system pCOLD I DNA. Their results indicated that the most thermolabile enzymes were *Pleos*-DyP1 and MnP2.

Contrary to the results reported by Fernández-Fueyo, et al., [7], the present research revealed that the *T*. *atroviride* DyP1 transformants were able to grow and completely decolorize the high redox-potential dye Reactive Black 5 (RB5), which can be explained as a result of both the use of an eukaryotic expression system and the cloning of *P*. *ostreatus* cDNA rather than a synthetic sequence.

While one of the most widely used hosts for the production of eukaryotic recombinant proteins is *Escherichia coli*, there are recurrent problems with recovering substantial yields of correctly folded and active enzymes [8]. *Trichoderma* species have been reported as good systems for the heterologous expression of proteins because they can carry out both eukaryotic post-translational modifications and efficient secretion [9].

It is known that DyP enzymes are significantly glycosylated, as is the case with BaDyPs, which are glycosylated with *N*-acetylglycosamine and manose [1]. Only through *in silico* analysis has it been possible to identify sites of glycosylation in the DyP of *P*. *ostreatus* [3,6–7], while a glycosylation percentage of 9 has been determined in the DyP of *P*. *sapidus* via the SDS-PAGE method [25]. Therefore, it is of great importance to obtain an expression system that ensures both the correct modifications and efficient secretion.

Several fungal DyPs have been expressed in *Trichoderma*, showing better results in terms of recovery and activity than those obtained with the DyPs expressed in *E*. *coli*. The identification and characterization of a DyP from *Pleurotus sapidus* were perfomed by Lauber, et al., [25], with this novel DyP sharing a high level of amino acid sequence homology (95%) with *P*. *ostreatus* DyP1. The DyP from *P*. *sapidus* (PsaDyP) was heterologously expressed in *Trichoderma reseei*. The highest enzymatic level activity obtained in the recombinant strain of *T*. *reseei* was 55,000 U/L. The recombinant PsaDyP was able to oxidize some classic peroxidase substrates, such as 2,2'-azino-bis(3-ethylthiazoline-6-sulfonate), substituted phenols such as 2,6-dimethoxyphenol, and the anthraquinonic dye Reactive Blue 5. In addition, it was also able to catalyze the bleaching of natural colorants such as β-carotene and annatto. This indicates that the PsaDyP has an oxidase function in addition to a peroxidase activity.

In this research, the heterologous expression of the *Pleos-dyp1* gene was achieved in the filamentous fungus *Trichoderma atroviride*, with the modified strains (TaDyP) able to decolorize mono-azo, di-azo, anthraquinone and anthracenedione dyes. DyP1 activity in the *T*. *atroviride* transformants was detected in submerged fermentation, while the TaDyP strains obtained decolorization percentages of 77 and 34% for AB129 and AYG (Fig 5), respectively, in submerged fermentations after 96 h. The present research found that the directed integration of the transformation vector pUE10 via homologous recombination in *T*. *atroviride* can prevent expression from being affected by modifications in the chromatin structure, subtelomeric silencing mutations or other phenomena [12].

The potential of DyPs of different origins for oxidizing various recalcitrant dyes continues to be an important research topic [7, 26–29]. Both the substrate preference of DyPs for anthraquinone dyes and their high level of peroxidase activity on a variety of organic compounds make them of great significance for biotechnological applications. The heterologous expression of eukaryotic proteins, such as Pleos-DyPs, in organisms such as *Trichoderma* can be used to enable correct folding and secretion with a native signal peptide or a specific signal peptide from *Trichoderma*. This can lead to the efficient production of active enzymes, which, in turn can favor not only the study, but also the production, of enzymes of interest to the field

## Supporting information

S1 Fig Dye decolorization during the submerged fermentation of *P*. *ostreatus*. The percentage decolorization of the dyes was monitored at the point of UV/Vis maximum absorbance for each dye during the growth of *P*. *ostreatus* via submerged fermentation in the presence of **(A)** AYG **(B)** RBBR and **(C)** AB129.
(TIF)
