# Correction: Differential regulation of *Pleurotus ostreatus* dye peroxidases gene expression in response to dyes and potential application of recombinant *Pleos*-DyP1 in decolorization

**DOI:** 10.1371/journal.pone.0213798

**Published:** 2019-03-07

**Authors:** The PLOS ONE Staff

The Funding section is incorrect. The complete, correct funding information is: “This work was supported by the Instituto Politécnico Nacional (IPN) Project No. SIP 20181110 (www.ipn.mx) and Comisión de Operación y Fomento de Actividades Académicas (COFAA-IPN) (https://www.cofaa.ipn.mx) which are gratefully acknowledged. JC-F was a postgraduate fellow with a CONACYT fellowship No. 589318 (http://www.conacyt.gob.mx).” The publisher apologizes for the error.

## References

[1] Cuamatzi-Flores J, Esquivel-Naranjo E, Nava-Galicia S, López-Munguía A, Arroyo-Becerra A, Villalobos-López MA, et al (2019) Differential regulation of *Pleurotus ostreatus* dye peroxidases gene expression in response to dyes and potential application of recombinant *Pleos*-DyP1 in decolorization. PLoS ONE 14(1): e0209711 10.1371/journal.pone.0209711 30608975 PMC6319807

