# Supplementary figures and images for: Differential regulation of Pleurotus ostreatus dye peroxidases gene expression in response to dyes and potential application of recombinant Pleos-DyP1 in decolorization
Source: PLoS One. 2019 Jan 4;14(1):e0209711. doi: 10.1371/journal.pone.0209711 (PMC6319807; doi:10.1371/journal.pone.0209711)

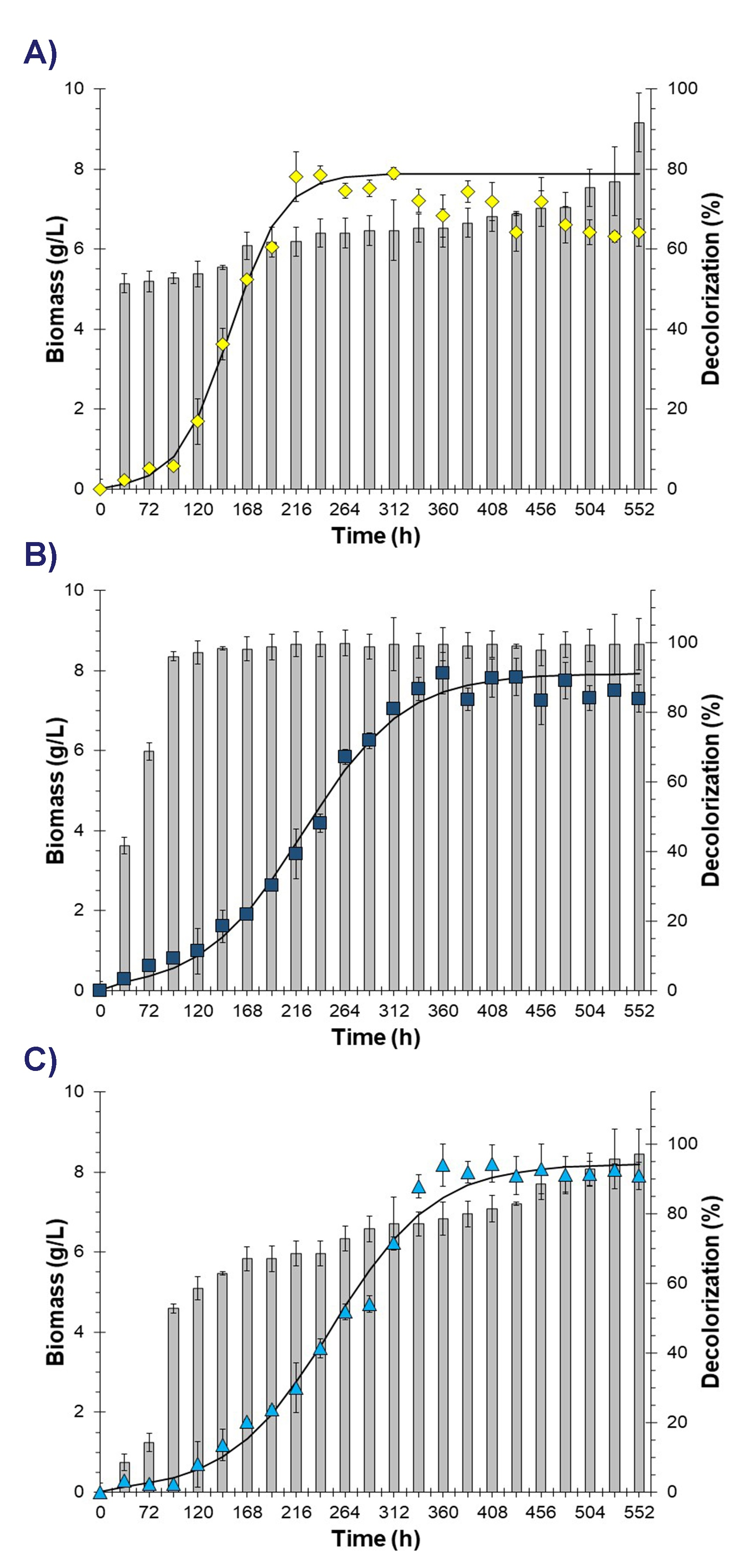

Supplement: S1 Fig — The percentage decolorization of the dyes was monitored at the point of UV/Vis maximum absorbance for each dye during the growth of P. ostreatus via submerged fermentation in the presence of (A) AYG (B) RBBR and (C) AB129. (TIF) [file pone.0209711.s001.tif]
